# Selection of new diagnostic markers for *Dirofilaria repens* infections with the use of phage display technology

**DOI:** 10.1038/s41598-022-06116-8

**Published:** 2022-02-10

**Authors:** Mateusz Pękacz, Katarzyna Basałaj, Alicja Kalinowska, Maciej Klockiewicz, Diana Stopka, Piotr Bąska, Ewa Długosz, Justyna Karabowicz, Daniel Młocicki, Marcin Wiśniewski, Anna Zawistowska-Deniziak

**Affiliations:** 1 Witold Stefański Institute of Parasitology, Polish Academy of Sciences, Warsaw, Poland; 2 Division of Parasitology, Department of Preclinical Sciences, Faculty of Veterinary Medicine, Warsaw University of Life Sciences-SGGW, Warsaw, Poland; 3 Division of Pathology, Department of Pathology and Veterinary Diagnostics, Faculty of Veterinary Medicine, Warsaw University of Life Sciences-SGGW, Warsaw, Poland; 4 Division of Pharmacology and Toxicology, Department of Preclinical Sciences, Faculty of Veterinary Medicine, Warsaw University of Life Sciences-SGGW, Warsaw, Poland; 5 Department of General Biology and Parasitology, Medical University of Warsaw, Warsaw, Poland

**Keywords:** Diagnostic markers, Parasitic infection

## Abstract

*Dirofilaria repens* is a parasitic nematode causing vector-borne disease (dirofilariasis), considered an emerging problem in veterinary and human medicine. Although main hosts are carnivores, particularly dogs, *D. repens* shows high zoonotic potential. The disease spreads uncontrollably, affecting new areas. Since there is no vaccine against dirofilariasis, the only way to limit disease transmission is an early diagnosis. Currently, diagnosis depends on the detection of microfilariae in the host bloodstream using modified Knott's test or multiplex PCR. However, the efficacy of tests relying on microfilariae detection is limited by microfilariae periodic occurrence. Therefore, a new reliable diagnostic test is required. Our study aimed to select new diagnostic markers for dirofilariasis with potential application in diagnostics. We focused on single epitopes to ensure high specificity of diagnosis and avoid cross-reactivity with the other parasite infections common in dogs. Using phage display technology and 12-mer peptides library, we selected epitopes highly reactive with IgG from sera of infected dogs. Additionally, our study presents the possibility of detecting *D. repens* specific cell-free DNA in dogs with no microfilaria but high IgG and IgM antibody levels against parasite somatic antigen.

## Introduction

*Dirofilaria repens* is a causative agent of subcutaneous dirofilariasis − a widespread mosquito-borne zoonosis. Infections occur prevalently in Europe, Southeastern Asia and occasionally in Africa. In recent years, disease spread primarily through Central and Eastern Europe countries: Poland, Slovakia or Ukraine^1^. According to the newest data, dirofilariasis reaches Northern Europe and Baltic countries, mainly Lithuania and Latvia. Autochthonous cases were also reported in Estonia and Finland^1^, while the first imported case was described in Denmark^2^. The spread of the disease depends on climate changes due to the introduction of more mosquitoes species able to transmit filariae. Also, human activity like travelling with pets increases the risk of transmission to new regions and contributes to the emergence of new endemic areas^1^.

Although main hosts are carnivores, especially dogs, parasites show high zoonotic potential. Human infections are described more frequently all over Europe^3^ and in rare non-endemic locations like Tanzania and other African countries^4^. Human infections were initially considered incomplete in the parasite life cycle; however, several patients with active microfilaremia were reported^5–9^. In the twenty-first century, over 70% of described human dirofilariasis cases were caused by *D.* *repens*, and 42.95% of recovered worms were mature, mainly females, and 26.42% contained microfilariae in the uterus^3^. These reports suggest that humans should also be considered as a potential reservoir of subcutaneous dirofilariasis.

Since no vaccine is available against *D.* *repens* infections, the only way to limit disease transmission is an early diagnosis. Currently, diagnosis depends on the detection of microfilariae in the host bloodstream using modified Knott's test or multiplex PCR^10,11^. Histochemical staining of acid phosphatase may complement Knott's method, enabling microfilariae differentiation^12,13^. However, available methods are ineffective in prepatent or occult (amicrofilaremic) infections and need specialized personnel and equipment, making them less widely accessible. The disease control is even more challenging as, in most cases, the infections are asymptomatic^14,15^ and may remain undiagnosed for years, being parasite reservoirs. Diagnosis of closely related *Dirofilaria* *immitis* is much easier since several diagnostic tests are available on the market^16^ and based on serological techniques detecting molecules secreted by parasites. However, knowledge about their specificity is inconsistent. Numerous researchers report cross-reactions with other dogs parasites despite indicated high specificity, e.g. *Angiostrongylus vasorum*, *Spirocerca lupi, Taenia taeniaeformis, Toxocara canis*, *Toxocara cati*, *Dipylidium caninum* and *D.* *repens*^17–20^; whereas some indicate their high specificity^21^. Furthermore, worm burden and sex of the parasites impact test sensitivity^22–24^, making it ineffective in prepatent and occult infections.

Our study aimed to select new diagnostic markers for subcutaneous dirofilariasis with potential application in the diagnostic test. We focused on single epitopes to provide high specificity of diagnosis and avoid cross-reactivity with other parasite infections common in dogs. Using phage display technology and 12-mer peptides library, we selected epitopes highly reactive with IgG and IgM antibodies from sera of infected dogs. Additionally, our study presents the possibility of detecting *D.* *repens* specific cell-free DNA in dogs with no microfilaria but high IgG and IgM antibody levels against parasite somatic antigen.

## Results

### Diagnostic examination

The first step in dogs classification was Knott’s test. Based on this method, dogs were initially distinguished as positive or negative. Then, sera from both positive and negative dogs were used in DrSA ELISA to evaluate IgG and IgM antibody response. Interestingly, despite the lack of microfilariae in the bloodstream, several dogs considered as non-infected showed high levels of IgG or IgM, which indicated false-negative results. We developed a new molecular detection method based on cell-free DNA presence detected by Real-Time PCR to confirm ELISA results. As cfDNA is known to degrade, we targeted three *Dirofilaria* genes (*16S rRNA*, *cox1* and *drpa*). Our approach enabled us to verify dogs negative in Knott’s test but with high OD in DrSA ELISA as positive but amicrofilaremic.

Interestingly, one dog was positive in the molecular test, although IgG and IgM levels were low. Targeted DNA fragments were amplified randomly and differed among examined individuals (Table 1). Fragments of internal control genes (*line1*, *gapdh*) were amplified in all analysed dogs sera samples (data not shown). The primers specificity was confirmed by reactions with genomic DNA isolated from common canine parasites (Table S1).

**Table 1:** Amplification pattern of targeted *D.* *repens* specific cfDNA fragments and OD values obtained in DrSA ELISA in microfilaremic (italic) and amicrofilaremic (bold) dogs.

Dogs	*s16*	*drpa*	*cox1*	OD IgG	OD IgM
**1**	−	−	+	1.13	0.62
**2**	−	+	+	1.51	0.85
**3**	−	+	−	0.90	0.63
**4**	+	+	+	0.84	0.89
**5**	+	+	+	1.54	0.47
**6**	+	−	+	0.60	0.35
**7**	+	−	+	0.98	0.24
**8**	+	+	−	0.97	0.62
**9**	−	−	+	1.27	0.41
*10*	+	+	+	1.13	0.54
*11*	+	+	+	0.83	0.45
*12*	−	+	−	1.47	0.97
*13*	−	+	+	0.80	0.32
*14*	+	−	+	1.36	0.90
*15*	+	−	N/A	1.35	0.52
*16*	−	+	−	0.57	0.59
*17*	+	+	N/A	1.08	0.64
*18*	+	+	N/A	1.49	1.38
*19*	+	+	+	1.04	0.32

“ + ” detected; “ − ” not detected; “N/A” not analyzed.

Finally, based on summarized results (Knott’s test, DrSA ELISA, Real-Time PCR), dogs were classified into three groups:

1. Microfilaremic (MF +)—dogs with microfilariae detected in the bloodstream using Knott’s test.
2. Amicrofilaremic (MF− DNA +)—dogs with no clear presence of microfilariae in the bloodstream but positive in Real-Time PCR analysis.
3. Non-infected (MF− DNA −)—dogs negative in both tests.

### *D. repens* somatic antigen (DrSA) ELISA enables to detect infection in microfilaremic and amicrofilaremic dogs

**Figure 1:**
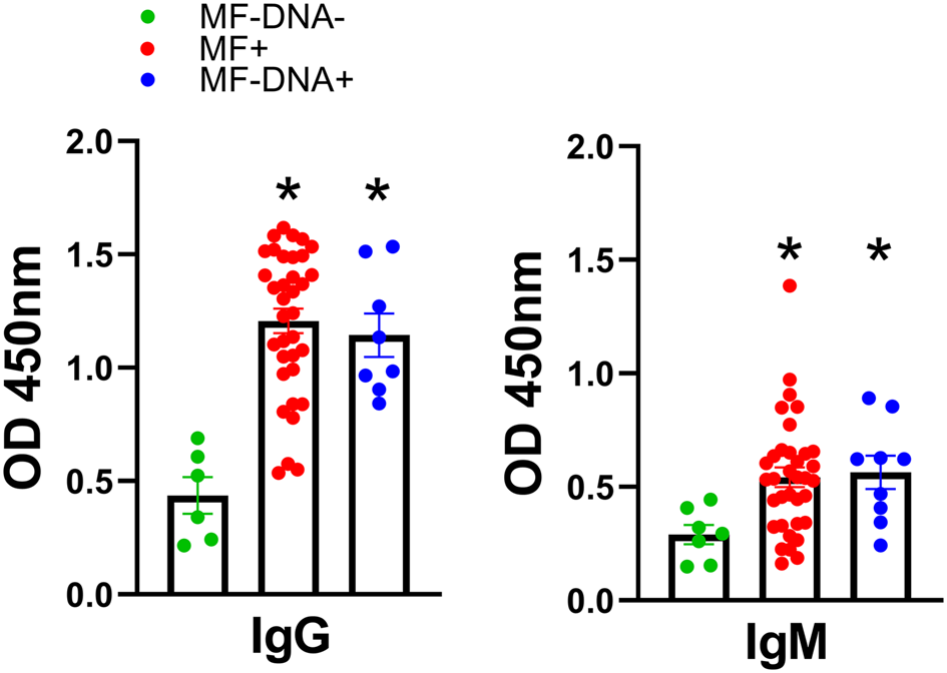
*D.* *repens* somatic antigen (DrSA) ELISA enables to detect infections in microfilaremic and amicrofilaremic dogs. The IgG and IgM levels were analyzed in dogs sera against DrSA. Plates were coated with 2.5 µg/ml of DrSA in carbonate buffer. The blocking step was performed with PBS containing 5% skimmed milk. Dog sera and secondary anti-dog IgG/IgM-HRP antibodies were used in 1:1,600 and 1:50,000 dilution, respectively. The statistically significant differences between examined groups are marked with an asterisk: *p < 0.05.

To analyze the IgG and IgM levels in dogs sera, we performed ELISA with adult somatic antigen (DrSA). Microfilaremic (MF +) and amicrofilaremic dogs (MF− DNA +) sera showed significantly higher IgG and IgM levels when compared to non-infected dogs (Fig. 1). However, in both positive groups (microfilaremic, amicrofilaremic), we identified several low responders with antibody levels comparable to non-infected dogs. Interestingly, there was no correlation between antibodies level and intensity of infection (number of microfilariae per ml of blood) (Figure S1 and S2).

### Screening of phage display peptide library enabled selection of 12 clones specific for both *D.* *repens* IgG and IgM antibodies

**Figure 2:**
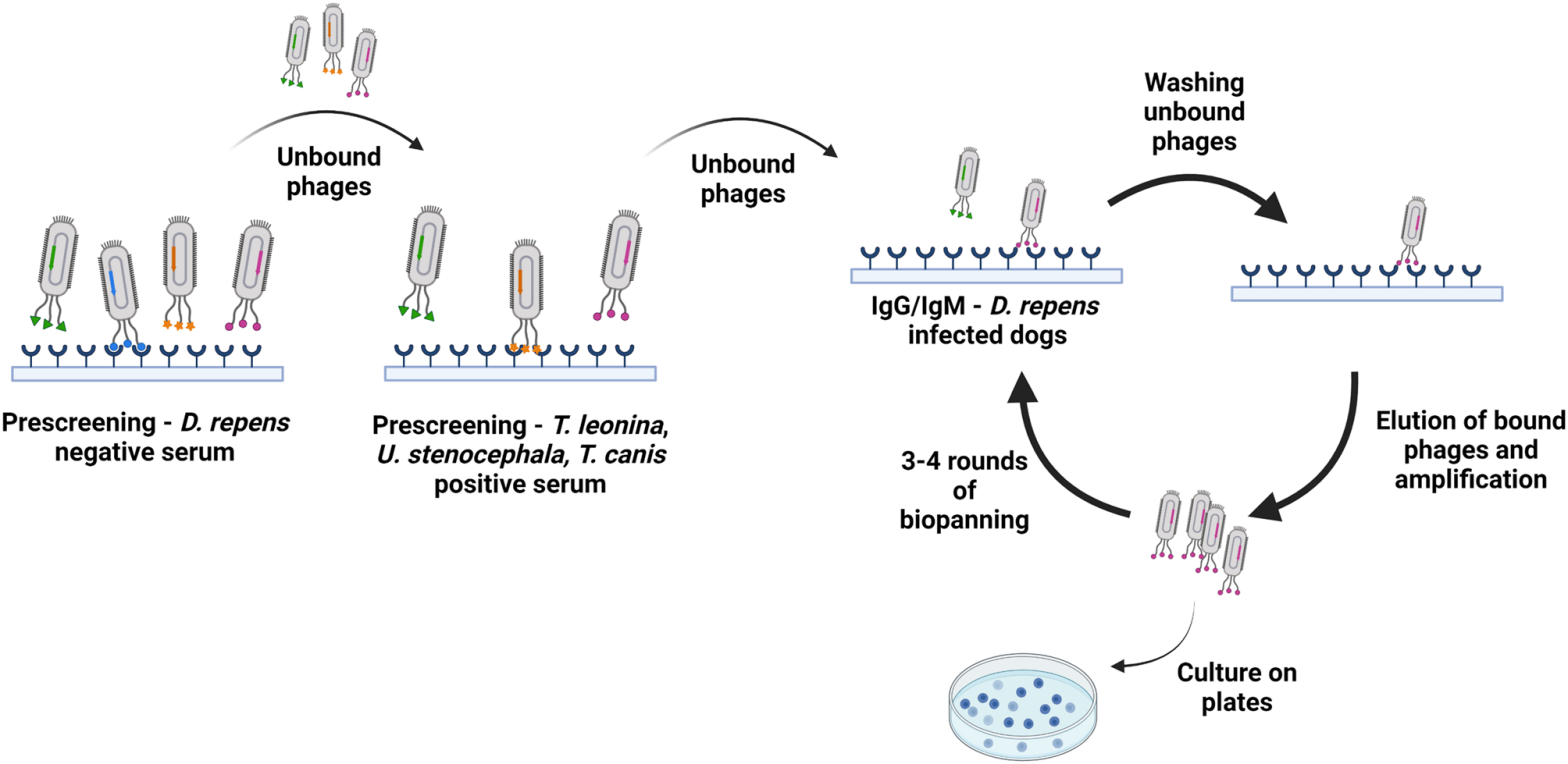
Screening of phage display peptide library enabled selection of 12 clones specific for both *Dirofilaria* *repens* IgG and IgM antibodies. Before actual biopanning, screening steps with sera from *D.* *repens* non-infected dogs, dogs infected with *T.* *leonina* and *U.* *stenocephala* and mice infected with *T.* *canis* were performed. After 3–4 rounds of actual biopanning with *D.* *repens* positive dogs sera 6 clones specific for IgG and 6 specific for IgM were selected. Figure created with BioRender.com.

Phage display technology was used to select new potential diagnostic markers for subcutaneous dirofilariasis. Briefly, technology is based on modified bacteriophages displaying short 12-mer peptides fused with coat protein. Biopanning of phage display peptide library was performed using IgG and IgM from dogs naturally infected with *D.* *repens* as a target. We added prescreening steps to reject non-specific clones and avoid cross-reactions with antibodies against other parasites molecules. First prescreening step was performed using sera from dogs free from *D.* *repens* infection, followed by sera from dogs infected with *T.* *leonina*, *U.* *stenocephala* and mice infected with *T.* *canis* (Fig. 2).

**Figure 3:**
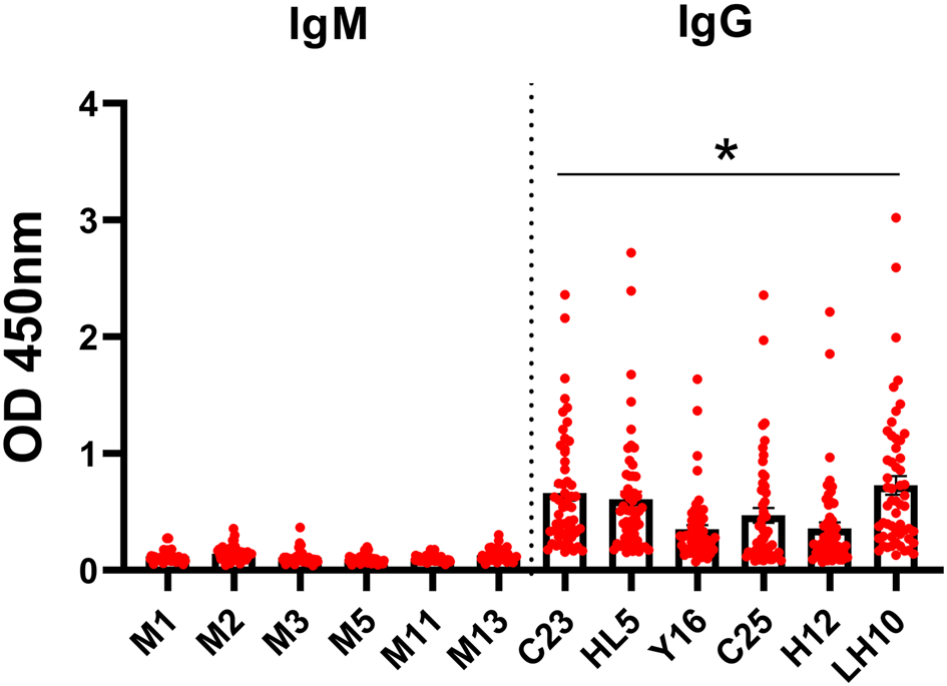
Clones specific for IgG showed higher reactivity with sera from dogs infected with *D.* *repens* than IgM-specific clones. To perform phage ELISA plates were coated with 1.46 × 10^11^ pfu/well of selected clones in TBS. The blocking step was performed with 0.1 M NaHCO_3_, 0.5% BSA and washed three times. Dogs sera were used in 1:800 dilution in blocking buffer, and secondary anti-dog IgG/IgM-HRP antibodies were diluted 1:50,000 in blocking buffer. The statistically significant differences between examined groups are marked with an asterisk: *p < 0.05.

Our approach enabled us to select 12-mer peptides specific for *D.* *repens* IgG and IgM antibodies. After four rounds of biopanning with IgG and three rounds with IgM, 96 and 48 clones, respectively, were picked from individual plaques, and DNA sequences were analyzed. After rejecting TUPs (Target Unrelated Peptides), the most abundant clones were selected: 6 clones potentially specific for IgG (C23, C25, LH10, HL5, Y16, HH12) and 6 clones potentially specific for IgM (M1, M2, M3, M5, M11, M13). Two peptides specific for IgG, C23 and LH10, appeared in 47/96 (~ 49%) and 26/96 (~ 27%) of screened clones, respectively. Selected clones were then tested in phage ELISA with sera from dogs infected with *D.* *repens*. The OD values of IgM clones were lower than IgG and were not considered in the experiments that followed (Fig. 3), while the three most reactive IgG clones (C23, HL5 and LH10) were further analyzed.

### LH10 clone revealed the highest diagnostic potential among examined clones

**Figure 4:**
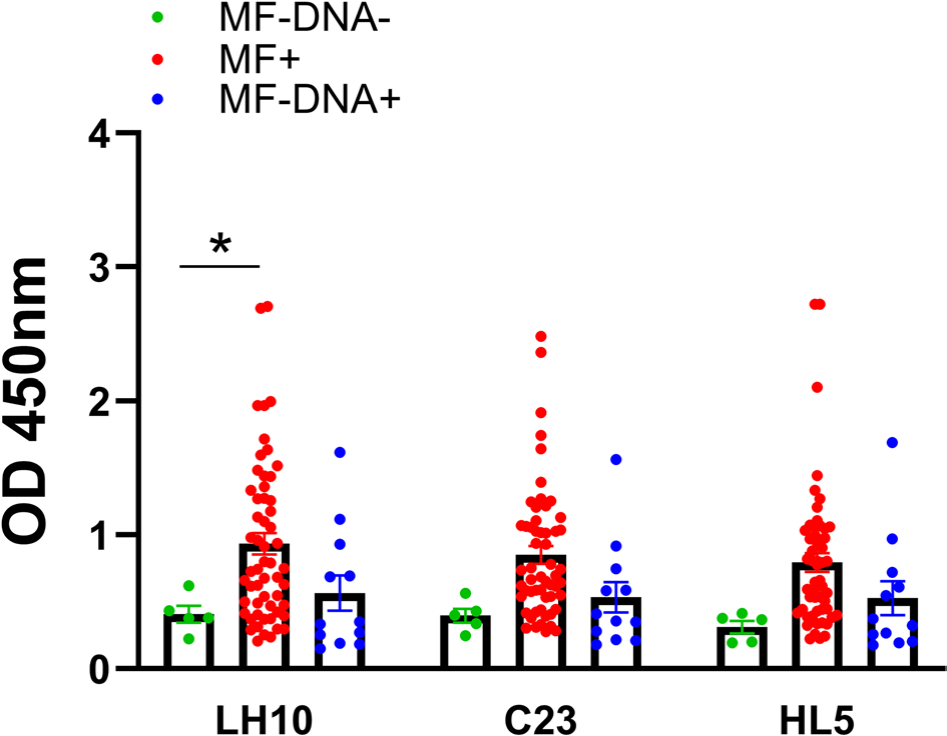
Phage ELISA showed that LH10 clone has the highest diagnostic potential among examined clones. The IgG responses to selected clones was evaluated in microfilaremic (MF +), amicrofilaremic (MF− DNA +) and negative (MF− DNA −) groups of dogs. Plates were coated with 1.46 × 10^11^ pfu/well of LH10/C23/HL5 clones in TBS. Plates were blocked with 0.1 M NaHCO_3_, 0.5% BSA and washed three times. Dogs sera were used in 1:800 dilution in blocking buffer. After six washes, secondary anti-dog IgG-HRP antibodies were used in a dilution of 1:50,000 in blocking buffer. The statistically significant differences between examined groups are marked with an asterisk: *p < 0.05.

Phage ELISA confirmed the diagnostic potential of C23, HL5 and LH10 clones with sera from *D.* *repens* microfilaremic, amicrofilaremic and non-infected dogs. Significantly higher levels of IgG from positive (MF +) dogs were observed for LH10 clone compared to non-infected (MF− DNA−) dogs. There were no statistically significant differences for clones C23 and HL5, despite increased IgG levels in the microfilaremic group. Additionally, neither of the peptides showed a significant difference between amicrofilaremic (MF− DNA +) and non-infected (MF− DNA−) dogs. Moreover, similarly to DrSA ELISA results, several individuals had IgG levels comparable to non-infected dogs (Fig. 4).

### LH10, C23 and HL5 clones are more reactive with microfilariae extract specific IgG

**Figure 5:**
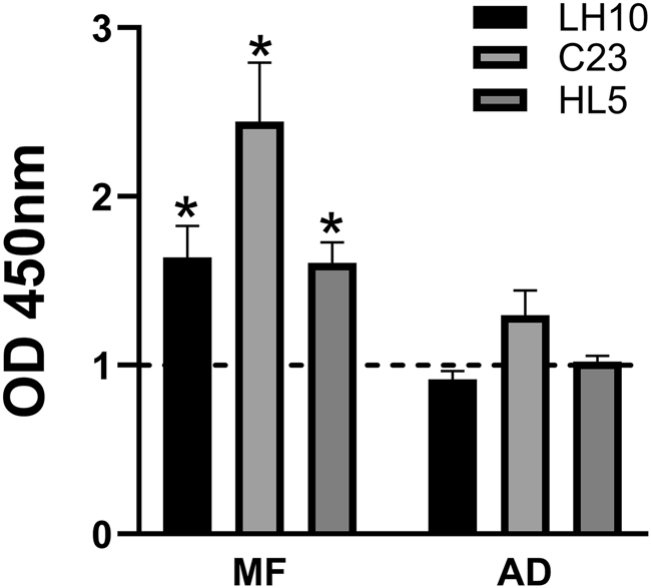
LH10, C23 and HL5 clones are more reactive with microfilariae extract specific IgG. IgG responses to selected clones were analyzed with the use of sera from rats immunized with *D.* *repens* adult worm (AD) and microfilariae (MF) extracts. Results are expressed as fold change IgG responses compared to control rat’s serum (dashed line). Plates were coated with 1.46 × 10^11^ pfu/well of LH10/C23/HL5 clones in TBS. Blocking step was performed in 0.1 M NaHCO_3_, 0.5% BSA and plates were washed three times. Rats sera were used in 1:800 dilution in blocking buffer. After six washes, secondary anti-rat IgG-HRP antibodies were used in a dilution of 1:5,000 in blocking buffer. The bars show mean ± SEM. The statistically significant differences between examined groups are marked with an asterisk: *p < 0.05.

The specificity of C23, HL5 and LH10 clones was confirmed in phage ELISA with sera from rats immunized with extracts from *D.* *repens* adult worm and microfilariae and lysis buffer as a control. Higher OD values were observed for all of the selected clones in microfilaria extract immunized rats (Fig. 5).

### “Wild type” vs. LH10 ELISA showed presence of IgG specific for LH10 peptide in sera from dogs infected with *D.* *repens*

**Figure 6:**
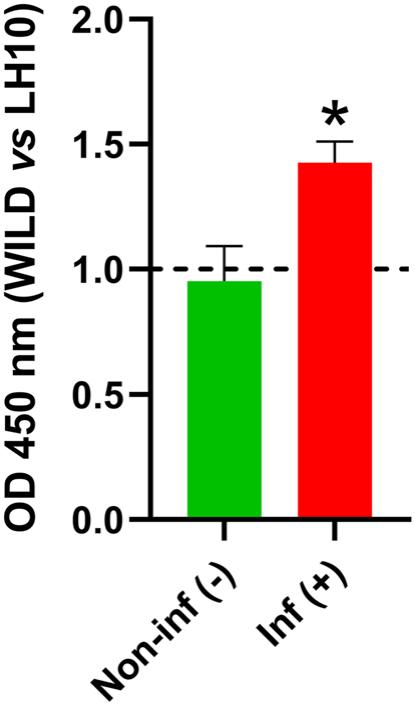
“Wild type” vs. LH10 ELISA showed the presence of IgG specific for LH10 peptide in sera from dogs infected with *Dirofilaria* *repens*. Levels of LH10-specific IgG in *D.* *repens* infected (red) and non-infected (green) dogs sera were compared to “wild type” M13 bacteriophage (dashed line). Plates were coated with 1.46 × 10^11^ pfu/well of LH10/“wild type” clones in TBS. The blocking step was performed in 0.1 M NaHCO_3_, 0.5% BSA and plates were washed three times. Dogs sera were used in 1:800 dilution in blocking buffer. After six washes, secondary anti-dog IgG-HRP antibodies were used in a dilution of 1:50,000 in blocking buffer. The bars show mean ± SEM. The statistically significant differences between examined groups are marked with an asterisk: *p < 0.05.

Based on the results obtained so far, the LH10 was identified as the most reactive and promising peptide. To exclude potential non-specific reaction of tested sera with native phage proteins, we designed phage ELISA with LH10 phage clone versus “wild” M13 phage (a clone with no library insert sequence). The OD values of IgG observed for LH10 clone were significantly higher than those observed for “wild” phage, suggesting the occurrence of antibodies specific for displayed peptides (Fig. 6).

## Discussion

Subcutaneous dirofilariasis is an emerging problem of human and veterinary medicine. Since there is no vaccine and current diagnostic methods are insufficient, zoonosis spread uncontrollably over the world. Developing a new reliable diagnostic tool, which could be applied not only by specialists but also by practitioners in every point of care like veterinary clinics, shelters could help control infections.

We selected epitopes highly reactive with IgG from the serum of dogs infected with *D.* *repens*. In addition, we demonstrated the possibility of detecting specific cell-free DNA in dogs with no microfilariae presence but with high IgG and IgM antibody levels against DrSA. First, using the DrSA ELISA test, we demonstrated that both IgG and IgM antibodies might be successfully used to diagnose both microfilaremic and amicrofilaremic/occult *D.* *repens* infections. This method allowed us to consider few individuals with no microfilariae in the bloodstream as a positive for *D.* *repens*, following additional confirmation by molecular approach. Such cases might indicate occult or prepatent infections.

*Dirofilaria* somatic antigens ELISA were successfully used in several previous studies^25–28^, and we showed it might be applied to diagnose cases without presence of microfilariae in the blood. The length of prepatent period varies depending on the study. Petry et al.^29^ reported that in experimentally infected dogs, prepatency lasts 169 to even 256 days post infection (dpi). In contrast, the others report patency beginning at 220–281 dpi, confirmed by molecular method and at 245–288 dpi established by Knott’s test^28^. Thus, in the first 5–9 months of infection, both described methods (Knott's and PCR tests) are ineffective. The use of serodiagnostics might be a better choice as antibody response starts in prepatent period and IgG levels constantly increase with the infection progress^26,28^.

In DrSA ELISA we identified several microfilaremic dogs with IgG and IgM antibody levels comparable to non-infected dogs. Interestingly, there was no correlation between the intensity of infection (number of microfilariae in the bloodstream) and levels of IgG and IgM. The explanation for low antibody titer is unclear, but we can hypothesize that it might be associated with the stage of infection and general medical condition of the host. In heartworm infections, stronger IgG response was showed in microfilaremic dogs than in amicrofilaremic^30^, however no evident correlation between antibody levels and number of microfilariae or adult worms was observed^31^. Simón et al.^30^ suggest relationship between IgG levels and host’s clinical status. A recent study shows that *D.* *repens* infection leads to anemia and a state of chronic stress response that likely affects antibodies' production^32^. It has also been reported that high microfilaremia develops in dogs with immunodeficiency-related conditions^33^. On the contrary, in dogs infected with *Brugia pahangi*, the IgG levels specific for adult worm extract were highest in amicrofilaremic dogs^34^. Similar trend was observed in human bancroftian filariasis, antibodies specific for sheath molecules were present in amicrofilaremic patients, with no response detected in patients with active microfilaremia^35^. The same significant inverse association was observed in Brugiosis^36^ and bovine filariae *Setaria digitata*^37^.

Our study aimed to select highly specific and immunoreactive markers of *D.* *repens* infections. To minimize the possibility of cross-reactions with antibodies against other parasite molecules, we focused on the short peptides using phage display technology. We screened the peptides library to select 12-mer epitopes reactive with *D.* *repens* IgG and IgM. Among all examined clones, LH10 had the highest diagnostic potential in the phage ELISA test. Evaluation of the “wild type” phage ELISA confirmed specificity to IgG antibodies present in infected dogs and analyses with rat sera indicated higher peptide reactivity with IgG against microfilariae extract. Results from phage ELISA shown a similar pattern to those from DrSA ELISA. Interestingly, several dogs still had low OD values for IgG levels comparable to non-infected dogs, which might be a limitation of the developed marker. However, further experiments with synthetic peptides will reduce the non-specific interaction of IgG with phage coat proteins which might increase the intensity of the signal.

Phage display is a high-throughput molecular screening technique used in multiple applications: epitope mapping^38,39^, searching for new ligands to target proteins^40^ or selection of antimicrobial/viral peptides^41,42^. Researchers recently successfully applied this technology in the parasitology field to select diagnostic markers, vaccine candidates, drug targets and to study general host-parasite interactions^43–46^.

The most challenging problem associated with serological diagnostic is cross-reactivity. Consequently, our limitation was the exclusion of the possible concomitant diseases or coinfections with other parasites. To reduce the risk of cross-reactivity, we performed additional prescreening steps with sera from dogs infected with *T.* *leonina* and *U.* *stenocephala* and mice experimentally infected with *T.* *canis*. Cross-reactions between molecules of the *Dirofilaria* genus were widely described in commercially available diagnostic tests and non-commercial ELISA tests. We plan to evaluate the cross-reactivity of selected peptides with the serum of *D.* *immitis* infected dogs in future studies.

In the present study, we identified specific *D.* *repens* cell-free DNA in sera samples from dogs naturally infected with *D.* *repens* and individuals with no microfilariae in the bloodstream. Analysis of 3 different genes revealed random amplification of targeted DNA fragments in both microfilaremic and amicrofilaremic individuals. Our results suggest that the pattern of released cell-free DNA may differ for each examined individual. The suggestion of random pattern release was previously reported by Ji et al.^47^ after sequencing of total plasma cfDNA from pooled 23 patients infected with *Echinococcus* spp. Parasite derived DNA was applied before in molecular detection of *Plasmodium*, *Leishmania*, *Schistosoma*, *Taenia* and *Wuchereria*^48–52^.

Cell-free DNA occurs in many body fluids like serum/plasma, urine or saliva, but might degrade quickly^53^. The knowledge about the source and kinetics of cfDNA remains puzzling. The mean half-time of circulating fetal DNA in maternal blood was established at 16.3 min (62) but may last several hours^54^. In the rabbit model of *S.* *japonicum*, depend on the monosexual or mixed sexual infections model, cfDNA was detectable in animals serum 3 and 7 weeks post infection, respectively. Nucleic acids release from inactive eggs lasted for more than 16 weeks^55^. Two mechanisms for release of cfDNA are possible: 1) passive—cfDNA derived from collapsed parasites tissues or cellular necrosis/apoptosis; 2) active—cfDNA is released directly from parasite or in excretory-secretory products^56^. According to Oi m. et al.^57^, in *D.* *immitis* infections cfDNA is derived mainly from microfilariae, whereas adult worms may be minor contributors. In research on *Brugia malayi*, cell-free DNA was detected in serum from jirds infected with *B. malayi* 56 days post infection^58^, which indicate that L3/L4 larvae may be considered as a main source of cfDNA during prepatent period. Despite some limitations caused by the physiological kinetics of cfDNA our technique may be regarded as a significant complement for serological diagnosis of subcutaneous dirofilariasis.

The present study demonstrated that DrSA ELISA might be an alternative diagnostic method for subcutaneous dirofilariasis infections. However, it requires constant access to *D.* *repens* parasites making it impossible to commercialize. We successfully implemented phage display technology to select new diagnostic markers. Our approach enabled us to search highly immunoreactive peptides with IgG from *D.* *repens* infected dogs. We consider LH10 peptide as a new diagnostic marker for subcutaneous dirofilariosis. However, further studies on synthetic peptides and a larger group of animals are required. Additionally, our study presents the possibility of detecting *D.* *repens* specific cell-free DNA in dogs with no microfilaria but high IgG and IgM antibody levels against parasite somatic antigen.

## Materials and methods

### Blood sample collection

All blood samples were collected in EDTA tubes from domestic and shelter dogs. One ml of blood was used in Knott's test, and remains were used to obtain plasma by centrifuging (15 min, 800 × g, 4 °C). Plasma samples were filtered through 0.22 µM syringe filters (Millex) to dispose of microfilariae and stored at − 70 °C until further analyses. All analyses were performed on the leftovers of blood samples collected during routine checkups or the diagnosis process by veterinarians in veterinary clinics or shelters in accordance with relevant guidelines and regulations.

### *D. repens* tissue lysates preparation

Adult *D. repens* worms were obtained from dogs scrotum during castration operations in veterinary clinics; microfilariae were isolated from blood using 5 μM filters (Whatman) and washed with PBS.

Adult and microfilariae lysates were prepared as we previously described^59^. Adult worms were washed with PBS to remove debris and homogenized manually in lysis buffer (8 M Urea, 40 mM Tris, 4% CHAPS). Approximately 160,000 microfilariae were suspended in lysis buffer and homogenized in TissueLyser (Qiagen, Hilden, NRW, Germany). Prepared homogenates were centrifuged for 15 min at 10,000 × g. The protein concentration in the supernatants was determined using BCA Protein Assay Kit (Pierce). The extract obtained from the adult parasite was termed *D.* *repens* Somatic Antigen (DrSA) and applied in the ELISA test with dog sera.

### Rat immunization and blood collection

Three-month-old male Wistar rats were immunized with an extract from *Dirofilaria repens* adult worms and microfilariae. Rats were subcutaneously administered with 100 µg of extract, followed by three doses of the booster: 75, 50 and 25 µg given on days 14, 28 and 42, respectively. Additionally, one rat was immunized with lysis buffer as a control group. Each of the doses was mixed in a 1:3 ratio with Imject™ Alum Adjuvant (Thermo Fisher Scientific). On day 49, rats were euthanized, and blood was collected in tubes with a clot activator. Blood tubes were centrifuged (15 min, 800 × g, 4 °C), and serum samples were stored at − 70 °C until further analyses. All experiments were performed in accordance with relevant guidelines and regulations. Ethical approval for this study was obtained from the 2nd Local Ethics Committee for Animal Experimentation, Warsaw University of Life Sciences-SGGW, Poland (approval number: WAW2/080/2019). The reporting in the manuscript follows the recommendations in the ARRIVE guidelines^60^.

### Dogs classification

All blood samples were tested with the use of Knott's test, DrSA ELISA and/or molecular method—Real-Time PCR. Based on summarized results, dogs were classified as microfilaremic, amicrofilaremic and negative.

### Modified Knott's test

One ml of blood was mixed with 9 ml of 2% formalin and centrifuged 10 min at 500 × g. The supernatant was discarded, and the pellet was stained with 1% methylene blue. One drop was placed on a microscope slide, covered with slip glass and observed under a light microscope. In positive samples number of microfilariae was counted and expressed in mf/ml.

### Molecular detection

Real-Time PCR was used to detect *D. repens* cell-free DNA (cfDNA) in plasma samples. cfDNA was isolated from 1 ml of filtered plasma using QIAamp Circulating Nucleic Acid Kit (Qiagen). Primers targeted at *D. repens drpa* (For_drpa 5' CGG AGG AAA TCA GAA TGA AAG TCG AAG 3'; Rev_drpa 5' CGT GCA TTC ATT GCC GCA TAG ATT TTA C 3'), *16S rRNA* (For_s16 5' GTG TGC TGC GCT ACA TCG ATG TT 3'; Rev_s16 5' ATA AAC CGC TCT GTC TCA CGA CG 3') and *cox1* (For_cox1 5' GTA GGT ATT GGT TCT TTG TTG GGT GCT A 3'; Rev_cox1 5' GTA ACA GCA GTA GAA CGC ATA TTC TGA GTA 3') genes were designed and used in qPCR reactions with isolated DNA as a template. Additionally, two pairs of primers specific for dogs *line1* (For_LINE1 5' C AAA TGC AAT GAA ACG CCG GG ACA 3'; Rev_LINE1 5' TCT TTC GTT GGA CAC CGA GG CTC 3')^61^ and *gapdh* (For_GAPDH 5' CAT GTT CCA GTA TGA TTC TAC CCA CG 3'; Rev_GAPDH 5' GGA GAT GGG ATT TCC ATT GAT GAC AAG 3')^62^ genes were used as a positive internal control. Reactions were performed according to a two-step procedure, including the dissociation curve step (Luminaris Color HiGreen qPCR Master Mix, high rox; Thermo Fisher Scientific) in a QuantStudio6 Real-Time PCR system (AppliedBiosystems) according to manufacturer’s protocol. After incubation for 2 min at 50 °C (Uracil-DNA Glycosylase pre-treatment) and 10 min denaturation step at 95 °C, 40 cycles of two-step amplification (15 s at 95 °C, 60 s at 60 °C) were performed. Data were collected during the annealing/extension step. The reaction volume of 10 μl contained 5 μl of 2 × Master Mix, 10 ng of isolated DNA and both forward and reverse primers in final concentration of 0.3 μM.

In addition, to confirm the specificity of primers targeted at *D.* *repens* DNA, the qPCR with 5 ng of genomic DNA isolated from common canine parasites (*Toxocara canis*, *Uncinaria stenocephala*, *Dipylidium caninum*, *Taenia krebbei*, *Mesocestoides litteratus*) was performed. Reactions conditions were as described above.

### *D. repens* somatic antigen ELISA

96-well half-area microplates (Corning) were coated with 2.5 µg/ml of DrSA in 0.1 M sodium carbonate buffer (pH 9.5) and incubated overnight at 4 °C. The plates were blocked with PBS containing 5% skimmed milk for 1.5 h at RT. All plasma samples were analyzed in dilution 1:1,600 in PBS, 1.5 h at RT. Anti-dog peroxidase-conjugated IgG and IgM (abcam, Table S2) were diluted 1:50,000 in PBS and incubated 1 h at RT. After every step, plates were washed three times with PBS containing 0.05% Tween-20. TMB substrate solution was added to the wells, developed at RT and stopped after 30 min with 2 M H_2_SO_4_. Optical densities were measured at 450 nm in a microplate reader (Synergy HT, BioTek).

### Biopanning with dog sera

Before each round of panning, five prescreening steps were undertaken. Ph.D.™-12 Phage Display Peptide Library (New England Biolabs) was first added to an empty well (1 h, RT) to eliminate polystyrene surface-binding peptides (PSBPs). Unbound phages were then transferred to subsequent wells for 1 h, RT: precoated with streptavidin (Pierce); coated with pooled sera from mice (diluted 1:100) infected with *Toxocara* *canis*^63^; coated with pooled dog sera (diluted 1:100) free from *Dirofilaria* infection and lastly, coated with 1:100 diluted serum from a dog infected with *Uncinaria* *stenocephala* and *Toxascaris* *leonina* (serum received from the Small Animal Hospital, Warsaw University of Life Sciences-SGGW). Unbound phages were collected from the last prescreening well and used for the actual biopanning.

The actual biopanning was conducted in a 96-well plate precoated with streptavidin (Pierce). Firstly, wells were washed 3 times with TBS, 0.5% Tween-20 (TBST) and coated overnight at 4 °C with 10 μg/ml biotin-SP anti-dog Fc IgG (Jackson ImmunoResearch) in 0.1 M NaHCO_3_. After 6 washes with TBST, wells were coated overnight at 4 °C with pooled sera in 1:100 dilution from dogs termed “low responders”. “Low responders” were microfilaremic dogs which sera, similarly to non-infected dogs had OD ≤ 0.7 in DrSA ELISA test).

After 6 washes with TBST, wells were blocked with 0.1 M NaHCO_3_, 3% BSA at 4 °C for two hours, followed by incubation with prescreened phages (overnight, 4 °C). After 10 washes with TBST, bound phages were eluted with 100 μl 0.2 M glycine–HCl pH 2.2 (15 min, RT with gentle agitation) and neutralized with 15 μl of 1 M Tris–HCl pH 9.1.

Amplification of phages for the next round was performed in *Escherichia coli* strain ER2738 (1:100 dilution of overnight culture with tetracycline) for 4.5 h, at 37 °C with shaking (250 rpm). The precipitation of phages from the supernatants was carried out overnight at 4 °C in the new tube in 1/6 volume of 20% PEG/2.5 M NaCl. After spinning the next day, the phage pellet was suspended in 1 ml of TBS and centrifuged again to pellet residual cells. The supernatant was transferred to a new tube, and the PEG/NaCl precipitation was repeated on ice for 30 min. After the last spin, the pellet was resuspended in 50 μl TBS and phage concentration was measured by titration and spectrophotometrically with the use of the following equation:

$$pfu{\text{/ml}} = \frac{{({\text{A}}_{269} - {\text{A}}_{320} ) \times 6 \times 10^{16} }}{7222}$$

The amplified eluate was used in the next round of panning after prescreening steps. In the second round, instead of overnight incubation at 4 °C, 2 × 10^11^ of phages were incubated on a plate for 1 h at RT and in the third and fourth round for 30 min. For rounds 1 and 3, blocking buffer with BSA was used (0.1 M NaHCO_3_, 3% BSA), and for rounds 2 and 4, 0.1 M NaHCO_3_, 3% non-fat dry milk was used.

For the fourth and last round of biopanning, pooled dog sera in 1:100 dilution defined as “high responders” were used. “High responders” were microfilaremic dogs which sera had OD ≥ 1.4 in DrSA ELISA test. In rounds 1–3 “low responders” sera were used to provide a high sensitivity of selected peptides and their usefulness in diagnosing dogs with low antibodies level against *D. repens* molecules. The use of “high responders” sera in the fourth round of biopanning was to confirm the specificity of screened peptides and select the most reactive ones. After elution of bound phages and neutralization, the phages were plated on LB/IPTG/X-gal plates for titering and plaques formation.

Approximately 100 blue plaques were picked for amplification and sequencing according to the manufacturer's protocol. SAROTUP software (http://i.uestc.edu.cn/sarotup3/index.html) was used for bioinformatic evaluation of obtained sequences to reject TUP (Target Unrelated Peptides) clones.

Additionally, we performed three rounds of biopanning with IgM antibodies from dogs infected with *D.* *repens* as a target in a separate experiment. As was shown in DrSA ELISA, IgM antibodies were less reactive than IgG (Fig. 1). Therefore, all three rounds of panning were undertaken using pooled sera in 1:100 dilution only from dogs with a high level of IgM antibodies (OD ≥ 0.8 in DrSA ELISA).

Prescreening, blocking (1 and 3 round: 0.1 M NaHCO_3_, 3% BSA; 2 round: 0.1 M NaHCO_3_, 3% non-fat dry milk) and washing steps, elution and amplification conditions and incubation times were the same as in the IgG screening experiment.

In contrast to previous actual biopanning, 96-well plate precoated with streptavidin (Pierce) was coated with goat anti-dog IgM Fc specific, Biotin conjugated (Agrisera) diluted 1:100 in 0.1 M NaHCO_3_. After three rounds of screening, eluted phages were plated, and 48 plaques were picked for amplification, sequencing and bioinformatic evaluation.

### Phage ELISA with dogs sera

According to NEB's protocol, selected phage clones were amplified in *E.* *coli* strain ER2738 and purified by PEG/NaCl precipitation method described above.

The 96-well half-area microplates (Costar) were coated with 1.46 × 10^11^ pfu/well in TBS of selected phage clone and incubated O/N at 4 °C with gentle agitation. The next day, plates were blocked with 0.1 M NaHCO_3_, 0.5% BSA for 2 h at 4 °C with gentle agitation and washed 3 times with TBST. The plates were incubated with dog serum samples in 1:800 and 1:1,600 dilutions in blocking buffer for 2 h at RT with gentle agitation. After 6 washes with TBST, the plates were incubated with goat anti-dog IgG/IgM-HRP antibodies (abcam) 1:50,000 in blocking buffer for 1 h at RT with gentle agitation. After 6 washes with TBST, 50 μl of TMB solution was added to the wells and plates were developed for 30 min. Optical densities were measured at 450 nm in a microplate reader (Synergy HT, BioTek).

Additionally, a “wild type” clone was amplified and included in the experiment to determine levels of antibodies that react with native phage molecules. Extra microplates were coated with 1.46 × 10^11^ pfu/well in TBS of “wild type” clone and incubated with dogs serum samples. All procedures were conducted as was described above. “Wild type” was a clone of M13 bacteriophage selected from peptide library (NEB) which did not display a 12-mer peptide fused to its coat protein.

### Phage ELISA with rats sera

To confirm the specificity of clones (C23, HL5, LH10), an ELISA test was performed with sera from rats immunized with extracts from *D.* *repens* adult worm, microfilariae and lysis buffer as control. All ELISA procedures were performed as was described in Sect. 2.9. Rats sera and secondary goat anti-rat IgG–HRP (Sigma) antibodies were used in 1:800 and 1:5,000 dilutions, respectively. Each sample was tested in triple technical repeats.

### Statistical analysis

All data are presented as mean ± standard error of the mean (SEM). Statistical analysis was performed using GraphPad Prism 8.0 (GraphPad Software, La Jolla, CA, USA) with unpaired *t*-test or one-way analysis of variance (ANOVA) followed by Fisher’s post-hoc test. Differences between groups were considered statistically significant at *p* < 0.05. Outliers were identified according to the two-standard deviation method. Spearman’s correlation coefficient was performed using R Statistical Software (R Foundation for Statistical Computing, Vienna, Austria) to analyze associations between number of microfilariae in the bloodstream and IgG/IgM levels.

## Supplementary Information

Supplementary Information.

## Data Availability

The data presented in this study are available on request from the corresponding author.
