# Supplementary material for: Selection of new diagnostic markers for Dirofilaria repens infections with the use of phage display technology
Source: Sci Rep. 2022 Feb 10;12:2288. doi: 10.1038/s41598-022-06116-8 (PMC8831495; doi:10.1038/s41598-022-06116-8)

Supplementary Material

Table S1. Ct values from Real-Time PCR with canine parasites genomic DNA.

gDNA	*s16*	*drpa*	*cox1*
Dirofilaria repens	13.50	19.02	13.67
Toxocara canis	-	-	-
Mesocestoides litteratus	-	-	-
Taenia krebbel	-	-	-
Dipylidium caninum	-	-	-
Uncinaria stenocephala	-	-	-

“-“ not detected.

Table S2. The list of antibodies and phage library used in experiments.

Product	Company	Catalog number
Goat Anti-Dog IgG H&L (HRP)	abcam	ab112852
Goat Anti-Dog IgM H&L (HRP)	abcam	ab112835
Goat Anti-Rat IgG Antibody, HRP conjugate	Sigma	AP136P
Biotin-SP (long spacer) AffiniPure Rabbit Anti-Dog IgG, Fc fragment specific	Jackson ImmunoResearch	304-065-008
Goat anti-Dog IgM Fc specific, Biotin conjugated	Agrisera	AS16 3129
Ph.D.™-12 Phage Display Peptide Library	New England Biolabs	E8111L

Figure S1. Spearman’s correlation coefficient was visualized between the number of microfilariae in the blood and IgM levels in dogs infected with *D. repens*; p-value = 0.8242; rho = -0.0415.

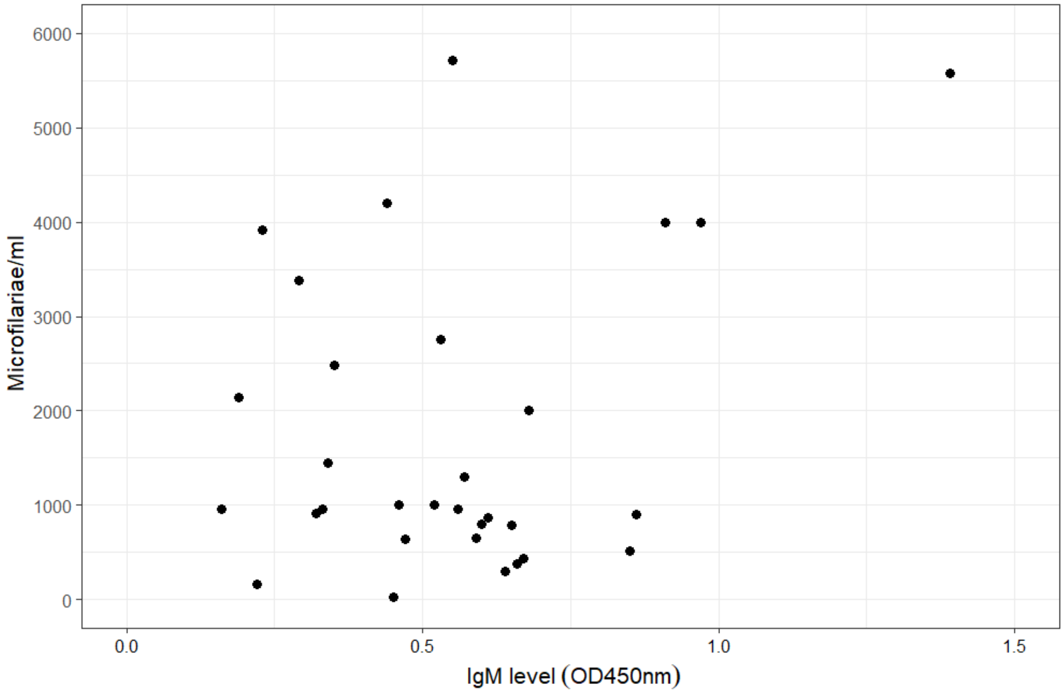

Figure S2. Spearman's correlation coefficient was visualized between the number of microfilariae in the blood and IgG levels in dogs infected with *D. repens*; p-value = 0.6605; rho = 0.0818.

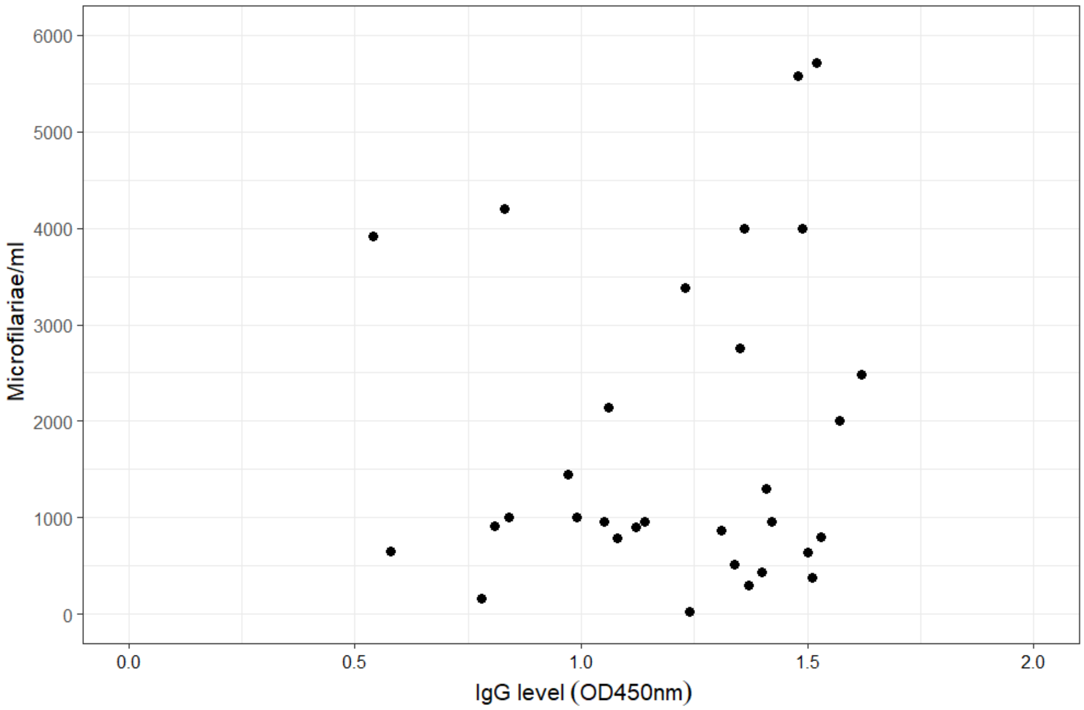

Supplement: Supplementary file 1 — Supplementary Information. [file 41598_2022_6116_MOESM1_ESM.pdf]
